# Androgen receptor–induced lncRNA SOX2-OT promotes triple-negative breast cancer tumorigenesis *via* targeting miR-320a-5p–CCR5 axis

**DOI:** 10.1016/j.jbc.2025.108428

**Published:** 2025-03-19

**Authors:** Yixuan Hu, Jin Bian, Weiwei Chen, Junfeng Shi, Xiaowei Wei, Yueyao Du, Wenwen Zhang

**Affiliations:** 1 Department of Oncology, Nanjing First Hospital, Nanjing Medical University, Nanjing, China; 2 Department of Medical Oncology of PLA Cancer Center, Jinling Hospital, Nanjing, China; 3 Department of Medical Oncology, Jinling Hospital, Affiliated Hospital of Medical School, Nanjing University, Nanjing, China; 4 Department of Breast Surgery, Renji Hospital, School of Medicine, Shanghai Jiao Tong University, Shanghai, PR China

**Keywords:** AR, CCR5, ceRNA, miR-320a-5p, SOX2-OT

## Abstract

Our previous study showed that androgen receptor (AR) promotes triple-negative breast cancer (TNBC) cell tumorigenesis, but the underlying mechanisms remain unclear. Herein, using microarray analysis of long noncoding RNA expression profiles, we identified an AR-related long noncoding RNA SOX2-OT in TNBC. We found that AR could promote TNBC tumorigenesis by acting as a transcription factor to activate the expression of SOX2-OT. Mechanistic analysis demonstrated that SOX2-OT serves as a molecular sponge for miR-320a-5p to regulate the expression of CCR5. In addition, SOX2-OT promotes TNBC cell proliferation and inhibits apoptosis in an miR-320a-5p-dependent manner. Using a xenograft mouse model, we found that SOX2-OT–CCR5 axis could promote TNBC tumorigenesis *in vivo*. Importantly, the AR–SOX2-OT–miR-320a-5p–CCR5 axis is manifested in the tissues of 165 TNBC patients. Collectively, our results suggest that SOX2-OT can regulate AR-induced TNBC tumorigenesis through the miR-320a-5p–CCR5 signaling axis and reveal the great potential of targeting SOX2-OT for the treatment of TNBC patients.

Triple-negative breast cancer (TNBC) has the worst prognosis compared with other subtypes of breast cancer (1). Due to its inherent aggressive clinical features and the lack of recognized therapeutic molecular targets, the regulatory mechanism of the malignant phenotype of TNBC has been a hot and difficult research topic (2). Currently, many studies on targeted therapy for TNBC are underway. The targets include vascular endothelial growth factor, PI3K, poly (ADP-ribose) polymerase, etc (3, 4, 5, 6). Androgen receptor (AR), a nuclear hormone receptor similar to estrogen receptor (ER), is expressed in 60% to 80% of breast cancer and 10% to 35% of TNBC patients (7). A systematic retrospective analysis found that AR is an independent prognostic factor for breast cancer. Progression-free survival and overall survival (OS) were significantly prolonged in patients with early stage breast cancer who had high expression of AR (8). Subgroup analysis revealed that this outcome was exclusively observed in ER-positive breast cancer patients, whereas it was absent in ER-negative breast cancer patients (8). Actually, the prognostic value of AR in TNBC is currently controversial. Another meta-analysis including 2826 TNBC patients showed that low AR expression is a high-risk factor for recurrence and death in TNBC (9). Conversely, it has also been reported in some studies that AR-positive TNBC patients have a lower survival rate (10, 11). Our published preclinical studies and other literatures have demonstrated that AR promotes TNBC cell tumorigenesis (12, 13, 14). However, the underlying mechanisms by which AR modulates the malignant phenotype of TNBC are poorly understood.

Long noncoding RNAs (lncRNAs), which are non–protein-coding transcripts more than 200 nucleotides in length, have been shown to play key roles in a wide range of biological processes, including cell proliferation, apoptosis, metastasis, differentiation, cell cycle arrest, development, and drug resistance (15, 16, 17, 18). It has been reported that lncRNAs can act as miRNA “sponges” that compete with miRNA-targeted mRNAs, thereby affecting miRNA-mediated gene regulation (19). For example, lncRNA nicotinamide phosphoribosyltransferase-AS acts as a competitive endogenous RNA (ceRNA) that rescues nicotinamide phosphoribosyltransferase degradation from miR-548b-3p to promote TNBC cell metastasis and regulate autophagy (20). Linc-ZNF469-3 serves as a sponge to target ZEB1 by sequestering miR-574-5p, thereby enhancing the invasiveness and stemness of TNBC and promoting lung metastasis (21). Our published study also showed that lncRNA ARNILA could promote SOX4 expression by acting as a ceRNA for miR-204, which ultimately leads to epithelial-to-mesenchymal transition, invasion, and metastasis of TNBC (22). The aim of this study was to investigate the role of lncRNAs in AR promotion of TNBC cellular tumorigenesis. We found that AR transcriptionally regulated the expression of lncRNA SOX2-OT, which is a molecular sponge of miR-320a-5p, thereby activating the CCR5 signaling pathway and promoting TNBC tumorigenesis.

## Results

### AR transcriptionally regulates lncRNA SOX2-OT expression in TNBC

**Figure 1:**
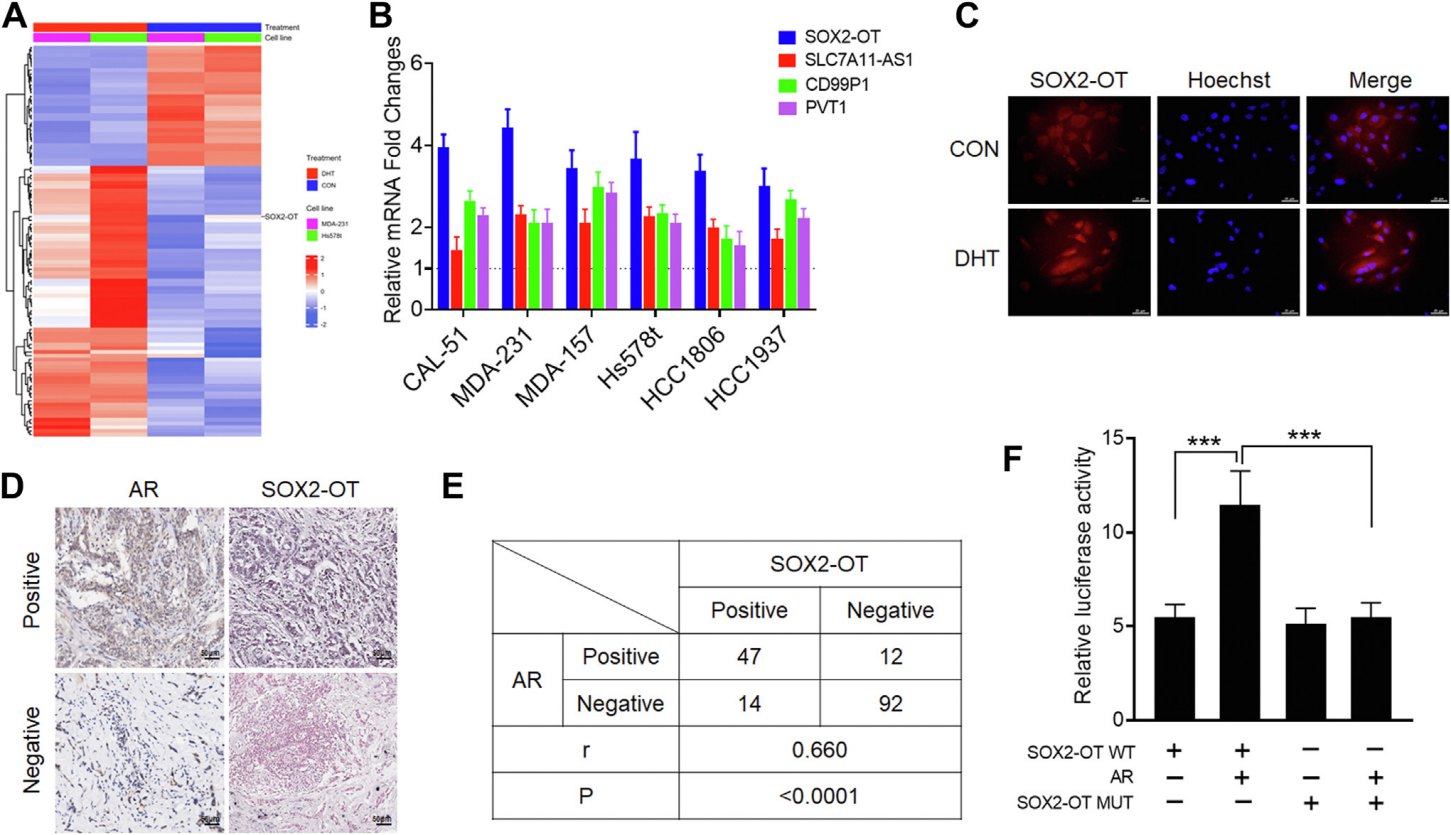
**AR transcriptionally regulates lncRNA SOX2-OT expression in TNBC.***A*, heat map representation of microarray data about the lncRNA levels in MDA-MB-231 and Hs578t cells treated with DHT. *B*, relative SOX2-OT RNA levels in the six TNBC cell lines treated with DHT. *C*, FISH images showing cellular localization of SOX2-OT in Hs578t cells treated with DHT. The scale bar represents 25 μm. *D*, representative IHC staining of AR and ISH staining of SOX2-OT in tissue of patients with TNBC. The scale bar represents 50 μm. *E*, correlation of AR and SOX2-OT expression in tissue from 165 patients with TNBC. *p* Values are calculated by Pearson correlation analysis. *F*, luciferase activity of SOX2-OT WT and SOX2-OT MUT upon transfection of AR plasmid in 293T cells. ∗∗∗*p* < 0.001 by *t* test. Results represented the average of three independent experiments, and the data represent the mean ± SD. AR, androgen receptor; DHT, dihydrotestosterone; IHC, immunohistochemistry; ISH, *in situ* hyridization; lncRNA, long noncoding RNA; TNBC, triple-negative breast cancer.

Our previous studies have shown that AR plays an important role in the progression of TNBC (13, 23, 24). To identify AR-related lncRNAs in TNBC, we treated two TNBC cell lines, MDA-MB-231 and Hs578t, with or without AR agonist dihydrotestosterone (DHT), then used microarray analysis to obtain the lncRNA expression profiles (Fig. 1*A* and Table S1). We chose four lncRNAs (SOX2-OT, SLC7A11-AS1, CD99P1, and PVT1), which were highest upregulated after DHT treatment in both TNBC cell lines. We next validated the mRNA expression level of these four lncRNAs in six TNBC cell lines treated with DHT and found that the expression level of SOX2-OT was most consistent with microarray analysis (Fig. 1*B*). FISH assays confirmed that SOX2-OT was located in both cytoplasm and nucleus of TNBC cells (Fig. 1*C*). It was worth noting that SOX2-OT expression was significantly altered by DHT (Fig. 1*C*). We next investigated the relationship between AR and SOX2-OT expression in the tissue of TNBC patients. Using immunohistochemistry (IHC) and *in situ* hyridization, the expression levels of AR and SOX2-OT were detected in a tissue microarray that contained tissue from 165 TNBC patients (25). Representative high-expression and low-expression AR and SOX2-OT immunostainings or hybridization fluorescence signal of TNBC samples are shown in Fig. 1*D*. Meanwhile, a significant positive correlation between AR and SOX2-OT expression was observed in those TNBC tissues tested (*r* = 0.660; *p* < 0.001; Fig. 1*E*). We then analyzed the mRNA expression levels of AR and SOX2-OT in the The Cancer Genome Atlas breast cancer patients. The results revealed that AR was positively correlated with SOX2-OT in the breast cancer populations (*r* = 0.317; *p* < 0.001; Fig. S1*A*).

Since AR has been reported to function as a transcription factor by binding to the target gene promoters (12), we investigated whether AR could transcriptionally regulate SOX2-OT. Bioinformatic analysis was used to predict transcription factor–binding sites in SOX2-OT promoters (Fig. S1*B*). We subcloned WT (SOX2-OT WT) and mutated (SOX2-OT MUT) AR binding site into dual-luciferase reporters. As shown in Fig. 1*F*, the relative luciferase activity of SOX2-OT WT was significantly enhanced after cotransfection of AR vector, whereas SOX2-OT mutant vector did not show a response to AR. Taken together, those results suggested that AR may be a transcription factor that promotes lncRNA SOX2-OT expression in TNBC.

### AR induces TNBC cell tumorigenesis *via* SOX2-OT

**Figure 2:**
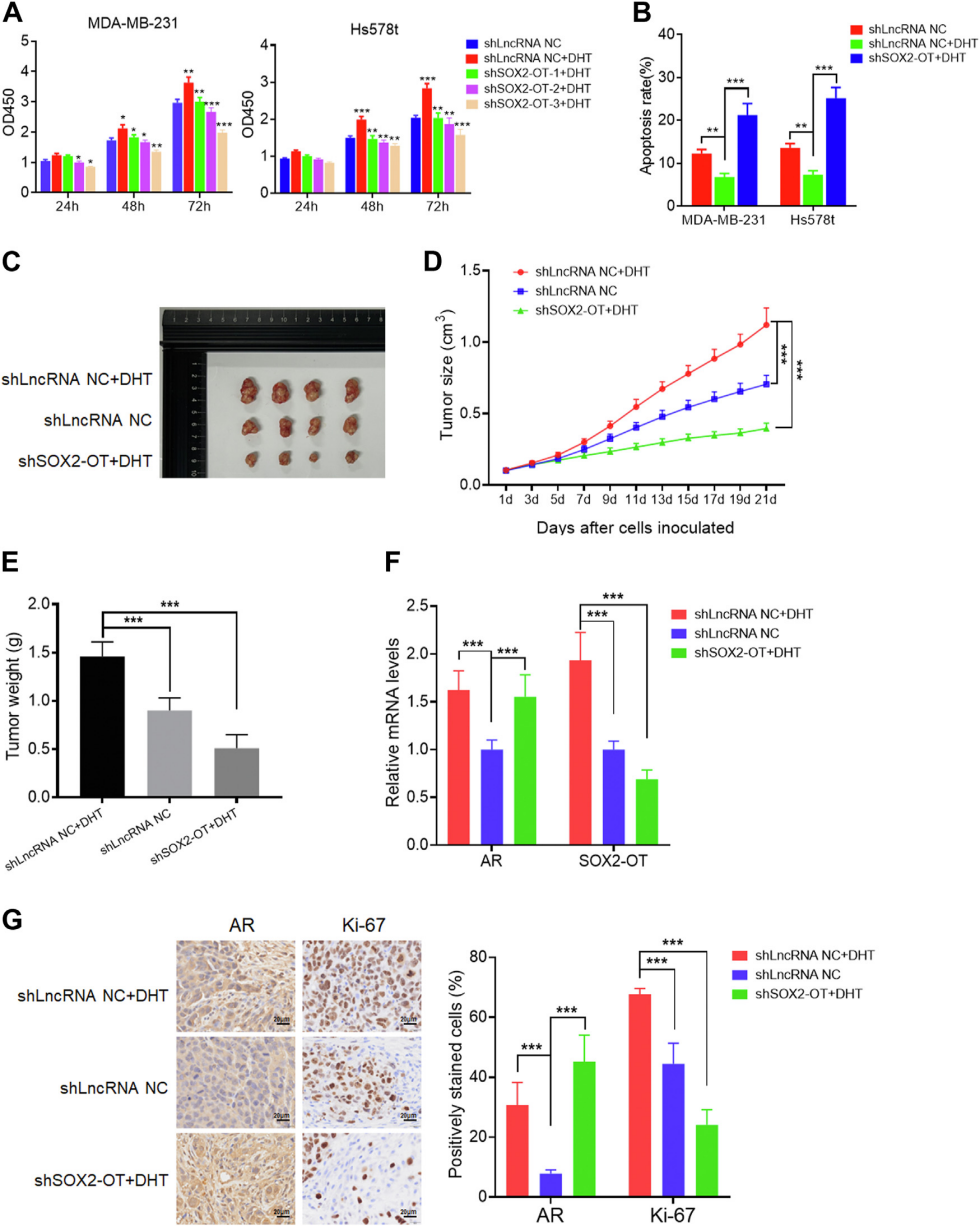
**AR induces TNBC cell tumorigenesis *via* SOX2-OT.***A*, cell viability assay of MDA-MB-231 and Hs578t cells transfected with three shSOX2-OTs or treated with DHT at various time points. The highest targeting efficiency for SOX2-OT shRNA (shSOX2-OT-3) was chosen for further studies. ∗*p* < 0.05, ∗∗*p* < 0.01, and ∗∗∗*p* < 0.001 by *t* test. *B*, cell apoptosis assay of MDA-MB-231 and Hs578t cells that were treated with shSOX2-OT or DHT. ∗∗*p* < 0.01, ∗∗∗*p* < 0.001 by *t* test. *C*, representative photographs of xenograft tumors were taken 3 weeks after injection. *D*, tumor sizes were measured at the indicated time points. ∗∗∗*p* < 0.001 by *t* test. *E*, excised tumors were weighed. ∗∗∗*p* < 0.001 by *t* test. *F*, relative mRNA expression levels of the excised xenografts. ∗∗∗*p* < 0.001 by *t* test. *G*, representative IHC staining in the tissue from the excised xenografts. The scale bar represents 20 μm. ∗∗∗*p* < 0.001 by *t* test. Results represented the average of three independent experiments, and the data represent the mean ± SD. AR, androgen receptor; DHT, dihydrotestosterone; IHC, immunohistochemistry; TNBC, triple-negative breast cancer.

To explore the role of SOX2-OT in AR-induced TNBC tumorigenesis, we treated MDA-MB-231 and Hs578t cells with shSOX2-OT and/or DHT and detected the changes of tumor cell biological characteristics. First, we confirmed that DHT treatment promotes SOX2-OT expression, which could be rescued by SOX2-OT knockdown (Fig. S2, *A* and *B*). Functional experiments showed that DHT treatment increased the cell growth of TNBC cells, which was reversed by SOX2-OT knockdown (Fig. 2*A*). We also noticed that DHT treatment could reduce TNBC cell apoptosis, whereas SOX2-OT knockdown increased the number of apoptosis cells in both TNBC cells (Figs. 2*B* and S2*C*). Next, we used a xenograft mouse model to further prove the role of SOX2-OT in AR-induced TNBC tumorigenesis *in vivo*. BALB/c nude mice were subcutaneously injected with shLncRNA negative control (NC)- or shSOX2-OT-transfected MDA-MB-231 cells and treated intraperitoneally with PBS or 7 mg/kg DHT every 2 days. As shown in Figure 2, *C*–*E*, DHT treatment was shown to strongly promote *in vivo* xenograft tumor growth, whereas SOX2-OT knockdown led to an inhibition of xenograft tumor growth (Fig. 2, *C*–*E*). Quantitative RT–PCR (qRT–PCR) confirmed that DHT treatment increased the mRNA expression levels of AR and SOX2-OT *in vivo* (Fig. 2*F*). Similarly, IHC staining assay displayed a consistent result with the qRT–PCR assay (Fig. 2*G*). We also noticed that DHT treatment increased the expression of proliferation index Ki-67, which was reversed by SOX2-OT knockdown (Fig. 2*G*). Collectively, these data suggested that AR could induce TNBC cell tumorigenesis *via* SOX2-OT.

### SOX2-OT acts as a molecular sponge for miR-320a-5p

**Figure 3:**
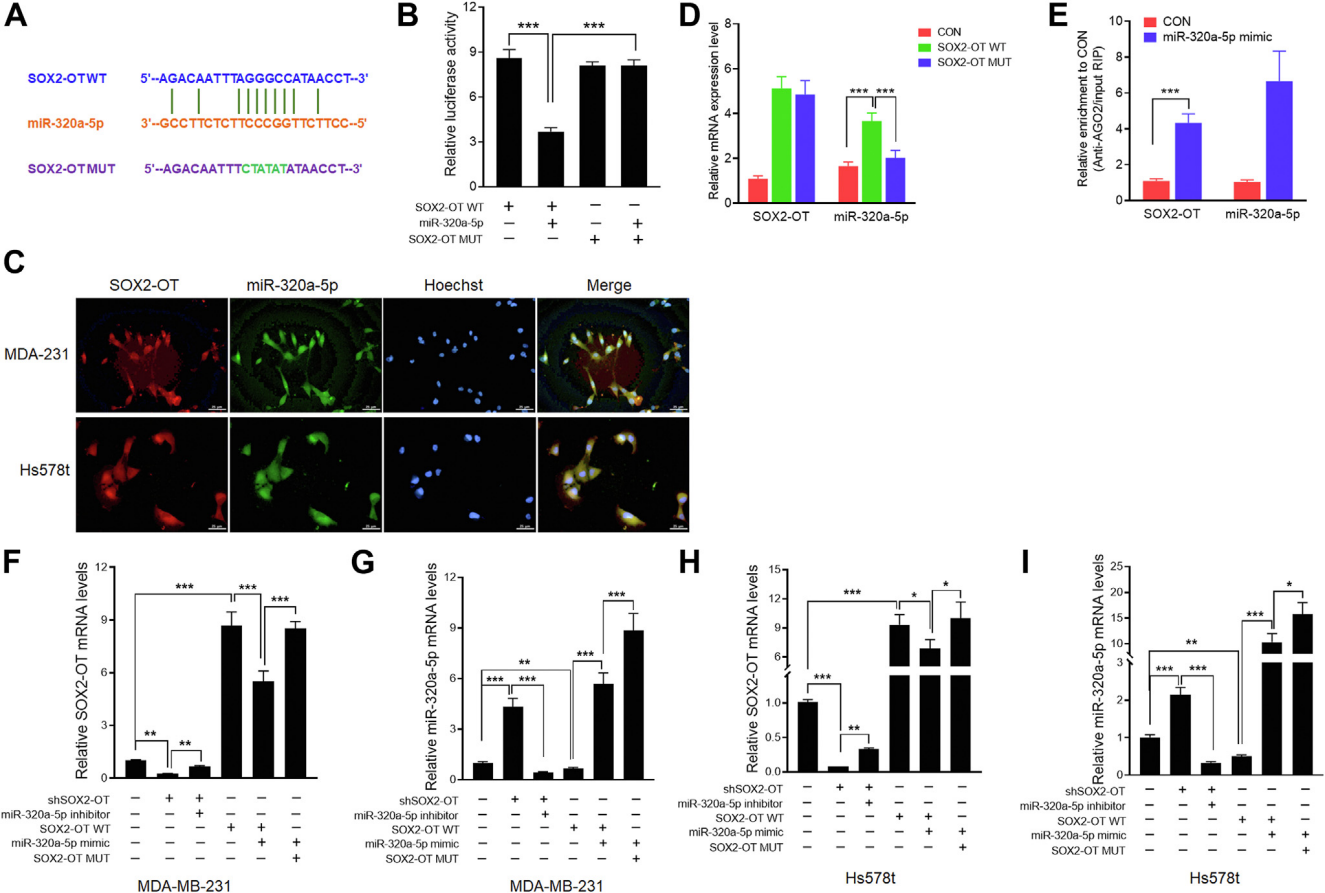
**SOX2-OT acts as a molecular sponge for miR-320a-5p.***A*, putative miR-320a-5p binding site and mutant sequences in SOX2-OT. *B*, luciferase activity of SOX2-OT WT and SOX2-OT MUT upon transfection of miR-320a-5p mimics in 293T cells. ∗∗∗*p* < 0.001 by *t* test. *C*, FISH images showing cellular localization of SOX2-OT and miR-320a-5p in MDA-MB-231 and Hs578t cells. The scale bar represents 25 μm. *D*, cell lysates of MDA-MB-231 transfected with SOX2-OT WT or SOX2-OT MUT were incubated with biotin-labeled SOX2-OT; after pull-down, mRNA expression levels of SOX2-OT and miR-320a-5p were detected by quantitative RT–PCR. ∗∗∗*p* < 0.001 by *t* test. *E*, anti-AGO2 RIP was performed in MDA-MB-231 cells overexpressing miR-320a-5p, followed by quantitative RT–PCR to detect SOX2-OT and miR-320a-5p associated with AGO2. ∗∗∗*p* < 0.001 by *t* test. *F*–*I*, relative SOX2-OT and miR-320a-5p levels in MDA-MB-231 and Hs578t cells transfected with SOX2-OT WT, SOX2-OT MUT, or shSOX2-OT, and miR-320a-5p mimic or inhibitor. ∗*p* < 0.05, ∗∗*p* < 0.01, ∗∗∗*p* < 0.001 by *t* test. Results represented the average of three independent experiments, and the data represent the mean ± SD. RIP, RNA immunoprecipitation.

Emerging studies have reported that lncRNAs could function as miRNA “sponges” in TNBC, competing with miRNA-targeted mRNAs and thus affecting miRNA-mediated gene regulation, referred to as ceRNAs (15, 19). To investigate whether SOX2-OT could act as a ceRNA to regulate TNBC tumorigenesis, we used bioinformatical analysis to predict potential miRNAs targeted by SOX2-OT in our previous study (26). We next performed dual-luciferase reporter assays to confirm the regulatory relationships between SOX2-OT and predicted miRNAs, miR-320a-5p (Fig. 3*A*). The result showed that cotransfection of miR-320a-5p significantly reduced luciferase activity of the reporter gene cloned with SOX2-OT WT but not mutant miR-320a-5p target sequence (Fig. 3*B*). Confocal microscopy of SOX2-OT and miR-320a-5p FISH showed the colocalization of SOX2-OT and miR-320a-5p in both cytoplasm and nucleus (Fig. 3*C*). To validate the direct binding of SOX2-OT to miR-320a-5p at the endogenous levels, we performed RNA-pull-down experiment by transfecting SOX2-OT WT or SOX2-OT MUT vectors in MDA-MB-231 cells. The results showed that the SOX2-OT WT group was able to successfully pull down miR-320a-5p, whereas the empty vector group and the SOX2-OT with mutations in miR-320a-5p targeting site group were not (Fig. 3*D*). To explore whether miR-320a-5p could regulate SOX2-OT in an AGO2-dependent manner, we performed anti-AGO2 RNA immunoprecipitation (RIP) in miR-320a-5p overexpressing MDA-MB-231 cells. We found that the traction of AGO2 on endogenous SOX2-OT was significantly enhanced in cells transfected with miR-320a-5p mimic (Fig. 3*E*). Moreover, our data showed that knockdown of SOX2-OT significantly elevated the level of miR-320a-5p, which was decreased by miR-320a-5p inhibitors (Fig. 3, *F*–*I*). Overexpression of SOX2-OT WT decreased the miR-320a-5p expression level, which was reversed by transfection of miR-320a-5p mimics. It is noteworthy that mutation of the miR-320a-5p binding site on SOX2-OT eliminated this reversal effect, further demonstrating that SOX2-OT serves as a molecular sponge for miR-320a-5p.

### SOX2-OT promotes CCR5 expression *via* miR-320a-5p

**Figure 4:**
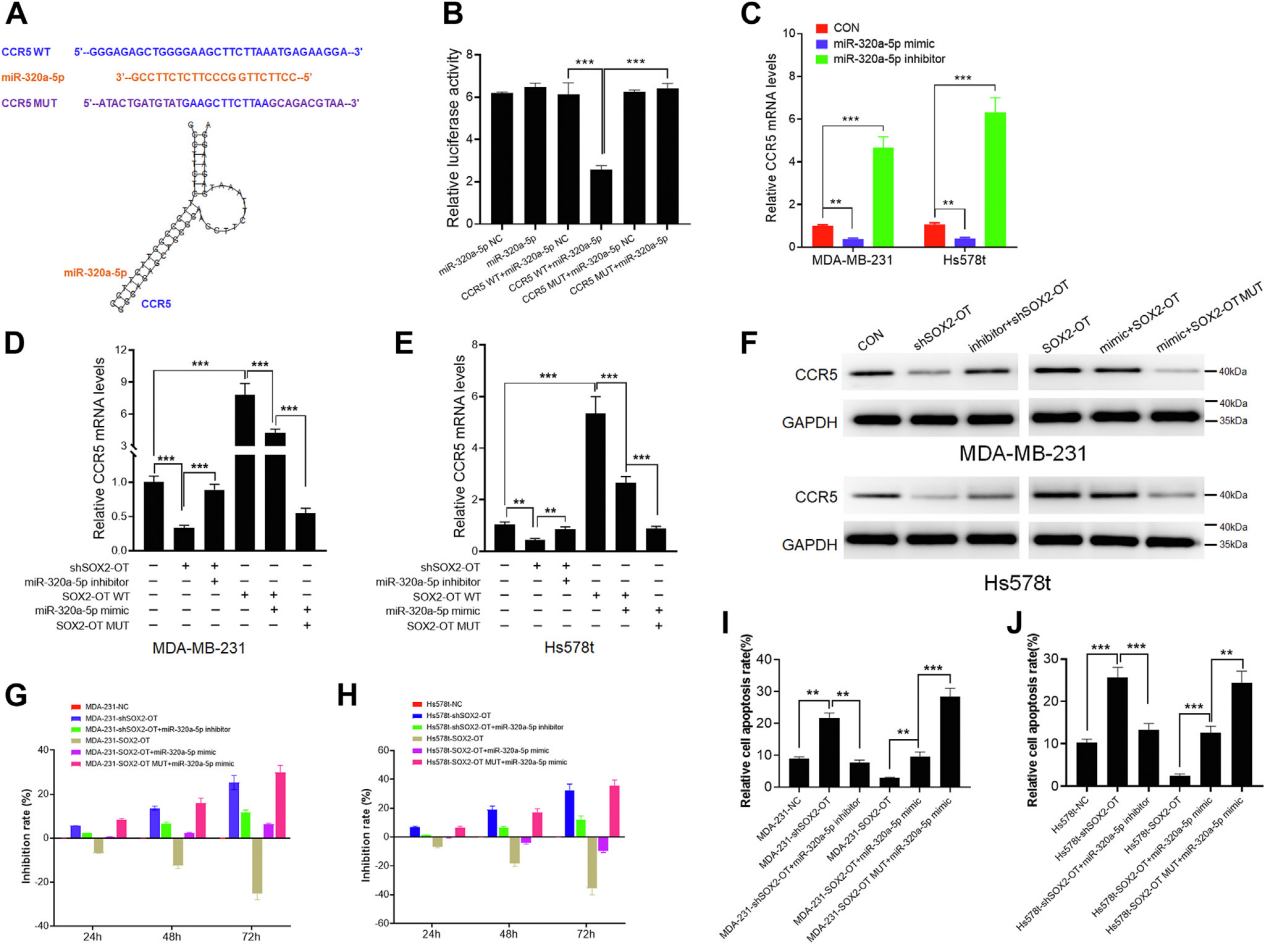
**SOX2-OT promotes CCR5 expression *via* miR-320a-5p.***A*, putative miR-320a-5p binding site and mutant sequences in the 3′UTR of CCR5. *B*, luciferase activity of CCR5 WT and CCR5 MUT upon transfection of miR-320a-5p mimics in 293T cells. ∗∗∗*p* < 0.001 by *t* test. *C*, CCR5 mRNA level of MDA-MB-231 and Hs578t transfected with miR-320a-5p mimic or inhibitor. ∗∗*p* < 0.01, ∗∗∗*p* < 0.001 by *t* test. *D* and *E*, CCR5 mRNA level of MDA-MB-231 and Hs578t transfected with SOX2-OT WT, SOX2-OT MUT, or shSOX2-OT, and miR-320a-5p mimic or inhibitor. ∗∗*p* < 0.01, ∗∗∗*p* < 0.001 by *t* test. *F*, relative protein levels of MDA-MB-231 and Hs578t transfected with SOX2-OT WT, SOX2-OT MUT, or shSOX2-OT, and miR-320a-5p mimic or inhibitor. *G* and *H*, cell growth inhibition rate of MDA-MB-231 and Hs578t cells transfected with SOX2-OT WT, SOX2-OT MUT, or shSOX2-OT, and miR-320a-5p mimic or inhibitor. *I* and *J*, cell apoptosis assay of MDA-MB-231 and Hs578t cells transfected with SOX2-OT WT, SOX2-OT MUT, or shSOX2-OT, and miR-320a-5p mimic or inhibitor. ∗∗*p* < 0.01, ∗∗∗*p* < 0.001 by *t* test. Results represented the average of three independent experiments, and the data represent the mean ± SD.

Using two online bioinformatical tools (mirDIP and miRWalk), we predicted that CCR5 is a potential target of miR-320a-5p. In order to verify the interaction between miR-320a-5p and CCR5, we transfected the WT (CCR5 WT) or mutant (CCR5 MUT) miR-320a-5p binding site into a dual-luciferase reporter (Fig. 4*A*). A significant decrease in the relative luciferase activity of CCR5 WT was observed after cotransfection of miR-320a-5p mimic, whereas the CCR5 mutant vector did not respond to miR-320a-5p mimic (Fig. 4*B*). Next, we examined CCR5 mRNA expression in the MDA-MB-231 and Hs578t cells after miR-320a-5p mimic or inhibitor treatment. We found that miR-320a-5p inhibitor increased the mRNA expression level of CCR5, whereas miR-320a-5p mimic significantly decreased CCR5 mRNA expression, suggesting that miR-320a-5p can target CCR5 in TNBC (Fig. 4*C*). To explore whether SOX2-OT was able to stimulate CCR5 expression by targeting miR-320a-5p, we examined CCR5 mRNA expression level in MDA-MB-231 and Hs578t cells after SOX2-OT overexpression or knockdown. As shown in Figure 4, *D* and *E*, we found that the mRNA level of CCR5 was significantly decreased after SOX2-OT knockdown, which was partially alleviated by miR-320a-5p inhibitor. Overexpression of SOX2-OT WT promoted the expression of CCR5, which was partially reversed by miR-320a-5p mimics treatment. Notably, the expression level of CCR5 was lower in the TNBC cells transfected with mutations in the miR-320a-5p-binding site on SOX2-OT (Fig. 4, *D* and *E*), suggesting that SOX2-OT induced the expression of CCR5 by competitively binding to miR-320a-5p. Subsequently, we detected the change of CCR5 protein expression level using Western blotting assay. The results showed consistency with the mRNA data (Fig. 4*F*). Taken together, our findings indicated that SOX2-OT could promote CCR5 expression *via* miR-320a-5p.

To explore whether SOX2-OT promoted TNBC cell tumorigenesis *via* miR-320a-5p, we assayed the proliferation and apoptosis of TNBC cells after the treatment of SOX2-OT and/or miR-320a-5p. It was noticed that the cell growth inhibition rate increased after SOX2-OT knockdown and decreased in SOX2-OT overexpressing cells at several different time points (Fig. 4, *G* and *H*). The miR-320a-5p inhibitor partially reversed the shSOX2-OT-induced cell growth inhibition, whereas the miR-320a-5p mimic reversed the cell proliferation of SOX2-OT WT treatment (Fig. 4, *G* and *H*). Of note, mutations in the miR-320a-5p-binding site on SOX2-OT further inhibited TNBC cell proliferation. Also, the results of cell apoptosis assay were consistent with those of Cell Counting Kit-8 assay (Fig. 4, *I* and *J*). Altogether, our findings suggested that SOX2-OT promoted TNBC cell proliferation and inhibited apoptosis in an miR-320a-5p-dependent manner.

### SOX2-OT–CCR5 axis promotes TNBC tumorigenesis *in vivo*

**Figure 5:**
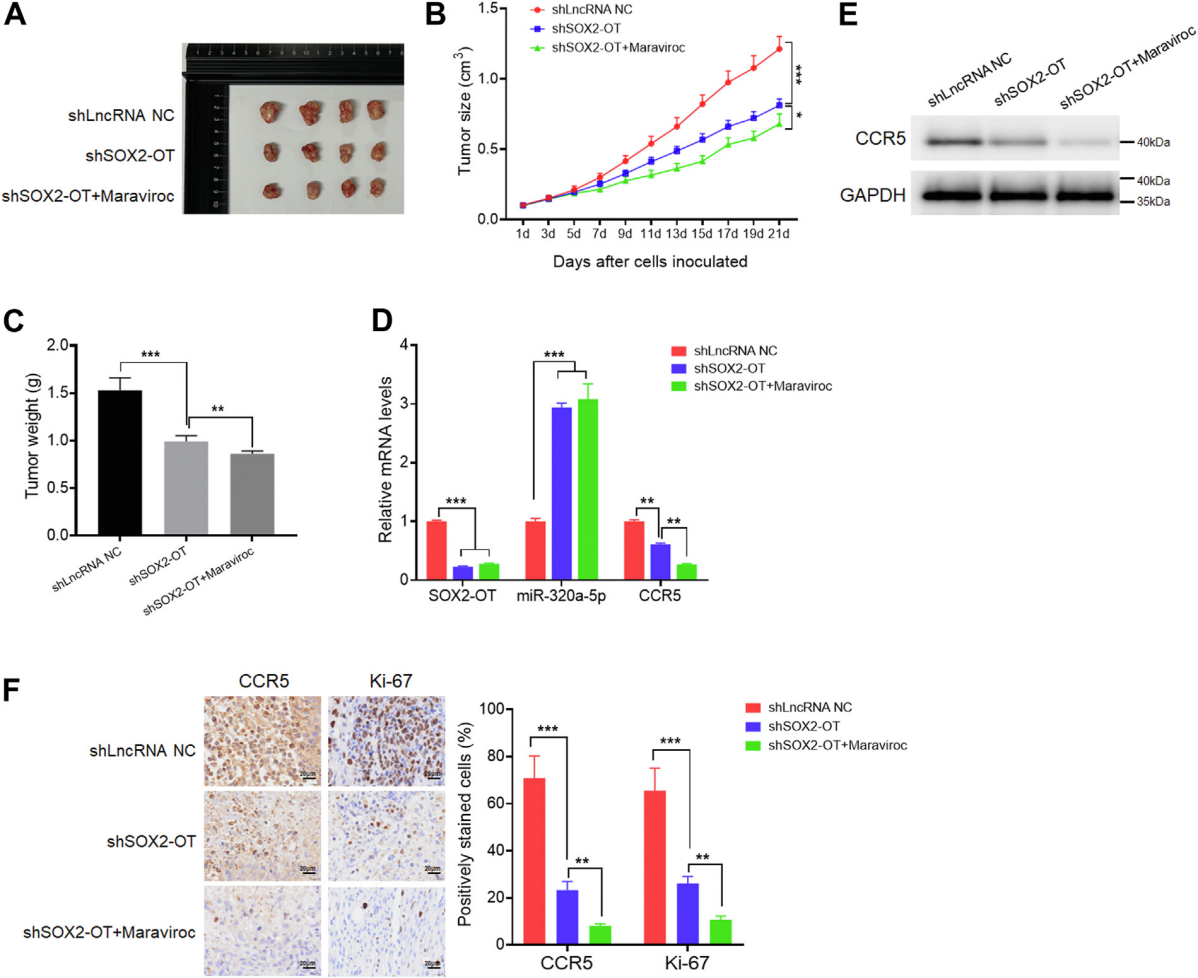
**SOX2-OT–CCR5 axis promotes TNBC tumorigenesis *in vivo*.***A*, representative photographs of xenograft tumors were taken 3 weeks after injection. *B*, tumor sizes were measured at the indicated time points. ∗*p* < 0.05, ∗∗∗*p* < 0.001 by *t* test. *C*, excised tumors were weighed. ∗∗*p* < 0.01, ∗∗∗*p* < 0.001 by *t* test. *D*, relative mRNA expression levels of the excised xenografts. ∗∗*p* < 0.01, ∗∗∗*p* < 0.001 by *t* test. *E*, relative protein expression levels of the excised xenografts. *F*, representative IHC staining in the tissue from the excised xenografts. The scale bar represents 20 μm. ∗∗*p* < 0.01, ∗∗∗*p* < 0.001 by *t* test. Results represented the average of three independent experiments, and the data represent the mean ± SD. IHC, immunohistochemistry; TNBC, triple-negative breast cancer.

To further prove the effect of the SOX2-OT–CCR5 axis in TNBC *in vivo*, we used a xenograft mouse model. Female BALB/c nude mice were subcutaneously injected with shLncRNA NC- or shSOX2-OT-transfected MDA-MB-231 cells, followed by intraperitoneally injection with PBS, or 10 mg/kg selective CCR5 antagonist maraviroc every 2 days. We found that SOX2-OT knockdown strongly inhibited the growth of xenograft tumors, and maraviroc treatment further enhanced this inhibitory effect (Fig. 5, *A*–*C*). In addition, the expression levels of SOX2-OT and CCR5 in SOX2-OT knockdown xenograft tumors were also significantly reduced, whereas miR-320a-5p levels were increased. Meanwhile, the expression level of CCR5 was further decreased after the addition of maraviroc (Fig. 5*D*). Western blotting and IHC staining assay showed that the expression levels of CCR5 and proliferation index Ki-67 were reduced in the shSOX2-OT-injected BALB/c nude mice. Maraviroc treatment further reduced their expression levels (Fig. 5, *E* and *F*). Collectively, these findings revealed that SOX2-OT–CCR5 axis could promote TNBC tumorigenesis *in vivo*.

### The expression of AR–SOX2-OT–miR-320a-5p–CCR5 axis in TNBC patients

**Figure 6:**
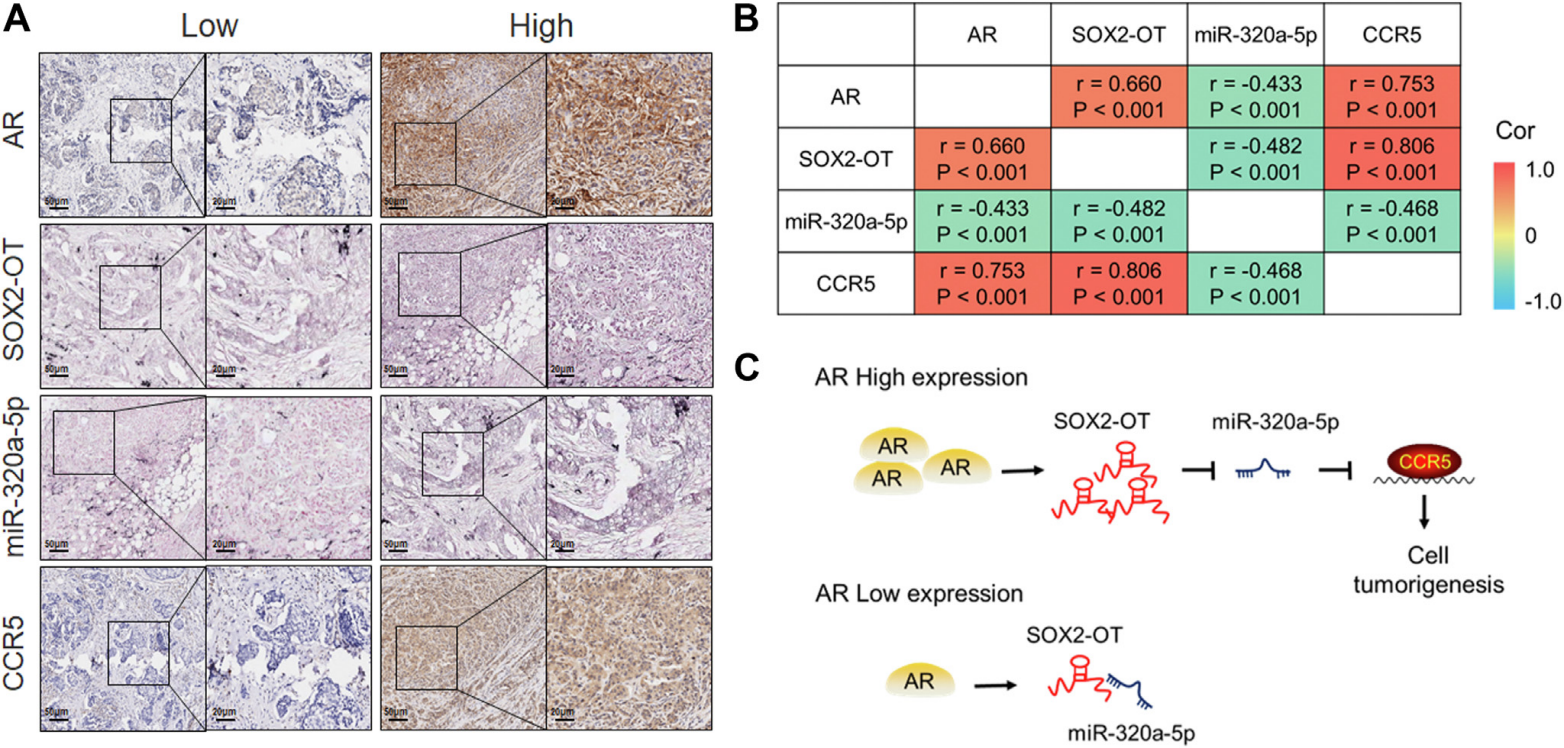
**The expression of AR–SOX2-OT–miR-320a-5p–CCR5 axis in TNBC patients.***A*, representative IHC or ISH staining of AR, SOX2-OT, miR-320a-5p, and CCR5 in the same tissue from patients with TNBC. The scale bar represents 20 μm (50 μm for enlarged diagram). *B*, correlation of AR, SOX2-OT, miR-320a-5p, and CCR5 expression in tissue from 165 patients with TNBC. *p* Values are calculated by Pearson correlation analysis. *C*, a schematic model of AR–SOX2-OT–miR-320a-5p–CCR5 signaling in the TNBC tumorigenesis. AR promotes SOX2-OT expression. In patients with a low AR expression, SOX2-OT blinds to miR-320a-5p, and the expression level of SOX2-OT is also low. In patients with a high AR expression, SOX2-OT acts as a molecular sponge for miR-320a-5p, leading to activating CCR5 signaling pathway and promoting TNBC tumorigenesis. AR, androgen receptor; IHC, immunohistochemistry; ISH, *in situ* hyridization; TNBC, triple-negative breast cancer.

To investigate the correlation between AR–SOX2-OT–miR-320a-5p–CCR5 axis in tissues of TNBC patients, we evaluated the expression of AR, SOX2-OT, miR-320a-5p, and CCR5 in tissue microarray specimens from 165 TNBC patients by using IHC and *in situ* hybridization. Representative staining is shown in Figure 6*A*, which showed that AR and CCR5 were simultaneously expressed in the same patient, whereas SOX2-OT and miR-320a-5p were expressed inversely. As shown in Figure 6*B*, a positive correlation between AR and SOX2-OT or CCR5 expression was identified by using the TNBC tissue array (*r* = 0.660, *p* < 0.001 and *r* = 0.753, *p* < 0.001, respectively). A significant positive correlation between SOX2-OT and CCR5 expression was identified (*r* = 0.806; *p* < 0.001). Meanwhile, we also found a negative correlation between miR-320a-5p and AR, or SOX2-OT, or CCR5 expression in tissue of 165 TNBC patients (*r* = −0.433, *p* < 0.001, *r* = −0.482, *p* < 0.001, and *r* = −0.468, *p* < 0.001, respectively; Fig. 6*B*). Taken together, our observations confirm the association between the expression of AR–SOX2-OT–miR-320a-5p–CCR5 signaling axis in TNBC patients.

## Discussion

AR has been well studied in prostate cancer, and inhibition of AR activity and androgen deprivation therapy are critical therapeutic tools in the treatment of metastatic prostate cancer (27). The AR antagonist, bicalutamide, has been approved by the US Food and Drug Administration in combination with luteinizing hormone–releasing hormone therapy for the treatment of advanced prostate cancer. AR also plays an important role in breast cancer, especially TNBC, and there is accumulating evidence that AR has the potential to be a therapeutic target for TNBC. Our previous study and other literatures had shown that AR promotes TNBC cell tumorigenesis (12, 13, 14). However, the underlying regulatory mechanisms by which AR promotes TNBC tumorigenesis are still unknown. In this study, our findings suggested that AR served as a transcription factor to promote the expression of lncRNA SOX2-OT, and SOX2-OT acted as a molecular sponge for miR-320a-5p, thereby activating the CCR5 signaling pathway and promoting TNBC tumorigenesis. Moreover, we also confirmed that the AR–SOX2-OT–miR-320a-5p–CCR5 signaling axis promoted TNBC tumorigenesis in a TNBC xenograft mouse model and TNBC patients.

AR is a member of the steroid hormone receptor family and is expressed in more than 70% of breast cancer patients (28, 29). The expression of AR has been reported to be a significant predictor of worse OS and disease-free survival in both univariate and multivariate analyses of TNBC patients (11). According to gene expression profiles, TNBC has been classified into six different subtypes, including basal-like 1 and 2, immunomodulatory, mesenchymal (M), mesenchymal stem-like, and luminal androgen receptor (10). The luminal androgen receptor subtype is characterized by high expression of AR as well as its downstream target genes and coactivators (10). The androgen testosterone is converted to DHT, which binds to AR in the cytoplasm and activates AR. The activated AR enters the nucleus and binds to the downstream promoter region of the target gene, regulating the transcription of the target gene and ultimately affecting the cell function. It has been shown that pharmacological blockade of AR, such as bicalutamide, inhibits the proliferation of TNBC cells, whereas AR agonist DHT is able to significantly promote the proliferation of TNBC cells (10, 30). Mechanically, our previous study demonstrated that DHT-induced AR activation inhibited G protein-coupled ER activation by directly binding to the promoter of G protein-coupled ER, thereby promoting cell growth in TNBC (13). AR also regulated the expression of cell cycle–related genes, including p73, p53, p21, and cyclin D1, through direct binding to the promoters of p73 and p21 in mesenchymal stem-like TNBC (30). In the present study, we aimed to elucidate other potential regulatory mechanisms by which AR facilitates TNBC tumorigenesis. We found that AR could transcriptionally regulate the expression of lncRNA SOX2-OT, which acts as a molecular sponge for miR-320a-5p, thereby activating the CCR5 signaling pathway and promoting TNBC tumorigenesis. CCR5 and its ligand CCL5 were found highly expressed in the basal and human epidermal growth factor receptor 2 genetic subtypes (31). Upregulation of CCL5 has been reported to increase the invasive potential of breast cancer cells (32). In prostate cancer, the CCL5–CCR5 axis has been reported to act as an upstream mediator to inhibit androgen–AR signaling (33). Therefore, the relevance and specific regulatory mechanisms of androgen–AR signaling and CCR5–CCL5 axis in breast cancer, especially TNBC, needs to be investigated in the future.

LncRNAs, non–protein-coding transcripts over 200 nt in length, have been found to play a wide range of functional roles in TNBC, including cell proliferation, apoptosis, metastasis, and drug resistance (15). The most common regulatory mechanism for lncRNAs is as ceRNA, in which lncRNAs act as miRNA "sponges," sequestering miRNAs and shielding their protein-coding counterparts from post-translational regulation (19). Our previous study has shown that lncRNA ARNILA can act as a ceRNA to regulate SOX4 through spongy miR-204, thereby promoting TNBC invasion and metastasis (22). Our another study found that lncRNA SOX2-OT is an oncogenic lncRNA that acts as a molecular sponge for miR-942-5p, which activates the PI3K–Akt signaling pathway and promotes TNBC metastasis *in vitro* and *in vivo* (26). SOX2-OT has been found to be aberrantly expressed in a variety of cancers, and high SOX2-OT expression is significantly associated with worsened clinical prognosis (34). Our published studies have also shown that in tissues from TNBC patients, high expression of SOX2-OT is positively correlated with poorer OS and distant metastasis-free survival and tends to correlate with poorer relapse-free survival (26). Thus, SOX2-OT may be a reliable prognostic biomarker and therapeutic target for TNBC patients. In this study, we identified a new regulatory mechanism of SOX2-OT, which mediates the role of AR in TNBC progression. Our results suggested that SOX2-OT can serve as a molecular sponge for miR-320a-5p, which ultimately promoted TNBC tumorigenesis *in vitro* and *in vivo* by activating the CCR5 signaling pathway. However, other potential regulatory mechanisms of SOX2-OT in TNBC progression need to be further investigated.

In summary, our findings constructed a schematic model that SOX2-OT could play a critical role in regulating AR-induced TNBC malignant phenotype through the miR-320a-5p–CCR5 signaling axis (Fig. 6*C*). We demonstrated that SOX2-OT can serve as a molecular sponge for miR-320a-5p, which ultimately promotes TNBC tumorigenesis by activating the downstream CCR5 signaling pathway. Our findings would certainly be helpful for understanding the pathogenesis of TNBC tumorigenesis and illustrating the great potential for developing SOX2-OT-targeted therapies in TNBC patients.

## Experimental procedures

### Cell lines and mice

TNBC cell lines, CAL-51, MDA-MB-231, MDA-MB-157, Hs578t, HCC1806, and HCC1937, were purchased from American Type Culture Collection. These TNBC cells were cultured in Dulbecco's modified Eagle's medium (GIBCO), RPMI1640, or McCoy's 5A. Female, 4- to 5-week-old BALB/c nude mice were purchased from Shanghai SLAC Laboratory animal Co Ltd. Experiments were conducted in accordance with the Helsinki Declaration and were approved by the Ethics Committee of Nanjing First Hospital.

### Clinical samples

Breast cancer tissue sections (HBreD075Bc01, 75 cancer cases and HBreD090Bc01, 90 cancer cases) were purchased from Outdo Biotech. All samples had a histologic diagnosis of invasive breast cancer with negative ER, progesterone receptor, and HER2.

### Reagents

AR agonist DHT (Aladdin; catalog no.: D413176) and selective CCR5 antagonist maraviroc (Aladdin; catalog no.: M125486) were protected from light and stored at −20 °C.

### Plasmids and transfection

The SOX2-OT small hairpin RNA (shSOX2-OT), nonspecific control shRNA, miR-320a-5p mimics, miR-320a-5p inhibitor, and their respective NC RNAs were all chemically synthesized by KeyGEN Biotech. Target sequences for shRNA and miRNA were provided as previously described (26). One with the highest targeting efficiency for SOX2-OT shRNA was chosen for further studies. Plasmids were transfected into cells according to the manufacturer’s protocol.

### Microarray analysis

MDA-MB-231 and Hs578t cells were treated with 100 nM DHT or vehicle for 48 h. The lncRNA expression profiling was performed on Arraystar Human LncRNA Microarray V3.0 platform (Agilent Technologies). The expression levels of all differentially expressed lncRNAs (fold change ≥2 and *p* ≤ 0.05) were plotted on a heatmap.

### Dual-luciferase reporter assays

Luciferase assay was performed as previously described (26). 293T cells were cotransfected with miR-320a-5p mimics and a luciferase reporter plasmid (GENE) carried WT or mutated SOX2-OT, AR, or CCR5 sequences. The Dual-luciferase assay system (Promega) was used to harvest cells according to the manufacturer’s instructions.

### RIP assay

RIP assay was performed as previously described (26). AGO2-specific and immunoglobin G antibodies were used for AGO2 immunoprecipitation according to the manufacturer’s instructions. The expression levels of SOX2-OT and miR-320a-5p were analyzed by qRT–PCR.

### RNA pull-down assay

SOX2-OT WT, SOX2-OT MUT, and lncRNA CON were transcribed in MDA-MB-231 cells, followed by biotin labeling using the Biotin RNA Labeling Mix (Roche) and T7 RNA polymerase (Roche). The transcripts were treated with RNase-free DNase I (Roche) and purified using the RNeasy Mini Kit (Qiagen). The whole-cell lysate was then incubated with the purified biotinylated transcripts for 1 h at 25 °C. Subsequently, the complexes were isolated using streptavidin agarose beads (Invitrogen), and the RNA complexes bound to the beads were collected. qRT–PCR was used to detect the expression levels of SOX2-OT and miR-320a-5p.

### Cell viability assay

Cell viability experiment performed used the Cell Counting Kit-8 in accordance with the recommended guideline (KeyGEN Biotech). Cell viability was analyzed as previously described (26, 35, 36, 37).

### Apoptosis analysis

Cells were harvested 48 h after plasmid transfection by trypsinization (without EDTA) and washed with PBS. Cell apoptosis was analyzed as previously described (36).

### Quantitative real-time PCR

Total cellular RNA was extracted using TRIzol (Invitrogen) and reversely transcribed according to the manufacturer’s instruction using the Step One System (Applied Biosystems, Life Technologies). Primer sequences (forward and reverse, respectively) were as listed below. AR: (forward) 5′-GGGCGAAGTAGAGCATCCT-3′, (reverse) 5′-GACGACCAGATGGCTGTCATT-3′; SOX2-OT: (forward) 5′-GAGGCTGGTGTAAGGCGATGTG-3′, (reverse) 5′-CATCCAAGGCACCGTGAATCCA-3′, miR-320a-5p: (forward) 5′-GCCTTCTCTTCCCGGTTCTTCC-3′, (reverse) 5′-GCGAGCACAGAATTAATACGACTCAC-3′, CCR5: (forward) 5′-GTCCTTCTCCTGAACACCTTCCA-3′, (reverse) 5′-GCAGTGCGTCATCCCAAGAG-3′, GAPDH: (forward) 5′-AGATCATCAGCAATGCCTCCT-3′, (reverse) 5′-TGAGTCCTTCCACGATACCAA-3′, U6: (forward) 5′-CTCGCTTCGGCAGCACA-3′, (reverse) 5′-TGGTGTCGTGGAGTCG-3′.

### Western blotting

RIPA buffer supplemented with protease and phosphatase inhibitors was used to extract total protein. The protein concentrations were determined using a BCA kit (Thermo Scientific). Antibodies used for Western blot were anti-CCR5 antibody (bs2514R; Bioss). Bands were normalized to GAPDH expression.

### Xenograft transplantation

Approximately 5.0 × 10^6^ MDA-MB-231 cells transfected with shLncRNA NC or shSOX2-OT were subcutaneously transplanted into the right side of the hind abdomen of nude mice. Tumor growth was checked every 3 days using vernier caliper. Tumor-loaded nude mice (n = 6 mice per group) were treated with PBS and maraviroc intraperitoneally every 3 days. About 3 weeks later, mice were euthanized and their tumors were excised.

### Immunohistochemistry

IHC was performed as previously described (36, 37). Antibodies used for IHC were anti-AR antibody (ab198394; Abcam), anti-CCR5 antibody (bs2514R; Bioss), and anti-Ki-67 antibody (ab16667; Abcam). Immunostained sections were scanned using a microscope (Aiovert 200; Carl Zeiss).

### FISH

Cells were fixed in 4% formaldehyde for 30 min at room temperature; washed with PBS 3 × 3 min; permeabilized in PBS containing 0.4% Triton X-100 for 15 min; washed with PBS 2 × 3 min and rinsed 3 × 5 min in 2× saline-sodium citrate buffer (SSC) prior to hybridization. Hybridizations were performed for 24 h at 42 °C in a humid chamber using anti-SOX2-OT or miR-320a-5p oligonucleotide probes conjugated to Alexa Fluor 488 (Invitrogen) or digoxigenin (DIG). Cells were washed 2 × 5 min in 2× SSC, 2 × 5 min in 1× SSC, 2 × 5 min in 0.5× SSC, and then 2 × 5 min in 0.1× SSC. The cells were restained with 5 μl Hoechst 33258 and imaged using a confocal laser-scanning microscope (Carl Zeiss).

### *In situ* hybridization

Paraffin-embedded tissue blocks were removed from the mouse xenograft model. Quantum dot FISH was performed using a DIG antibody–coupled quantum dot indirectly DIG-labeled oligonucleotide probe to detect the expression of SOX2-OT and miR-320a-5p.

### Statistical analysis

The data are represented as the means ± SD of three independent experiments. The log-rank test was used to evaluate the statistical significance of Kaplan–Meier plots. Student’s *t* test, log-rank test, Mann–Whitney *U* test, and Pearson correlation analysis were used for comparison. *p* Values <0.05 were deemed to be statistically significant: ∗*p* < 0.05, ∗∗*p* < 0.01, and ∗∗∗*p* < 0.001.

## Data availability

The datasets used and/or analyzed during the current study are available from the corresponding author upon reasonable request.

## Supporting information

This article contains supporting information.

## Conflict of interest

The authors declare that they have no conflicts of interest with the contents of this article.
