# Supplementary material for: Androgen receptor–induced lncRNA SOX2-OT promotes triple-negative breast cancer tumorigenesis via targeting miR-320a-5p–CCR5 axis
Source: J Biol Chem. 2025 Mar 19;301(4):108428. doi: 10.1016/j.jbc.2025.108428 (PMC12017981; doi:10.1016/j.jbc.2025.108428)
Supplement: Supporting Information [file mmc1.doc]

**Supplemental Figures and Table**

**
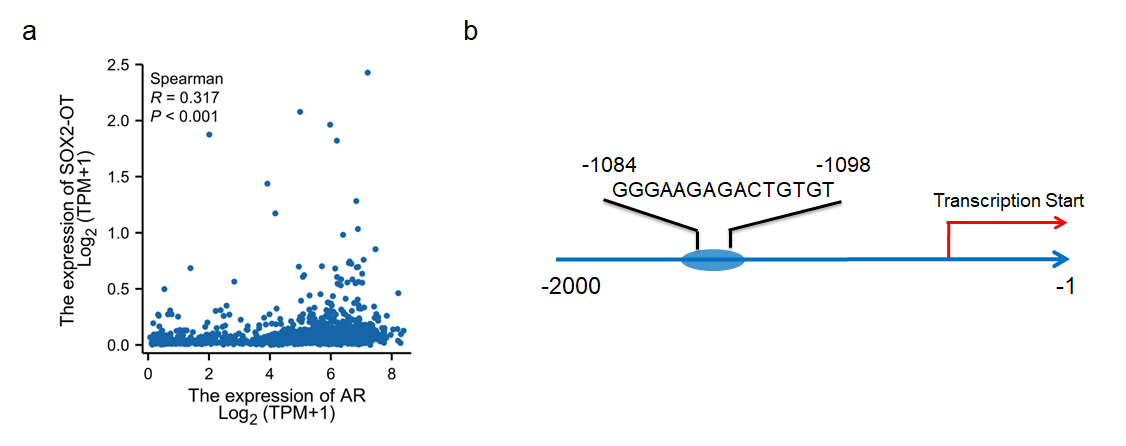
**

**Figure S1, related to Figure 1. AR promotes lncRNA SOX2-OT expression in TNBC.** a) Correlation between AR and SOX2-OT expression in TCGA breast cancer patients. P-values were calculated with Spearman correlation analysis. b) Potential binding sequences of AR in the SOX2-OT promoter region.

**
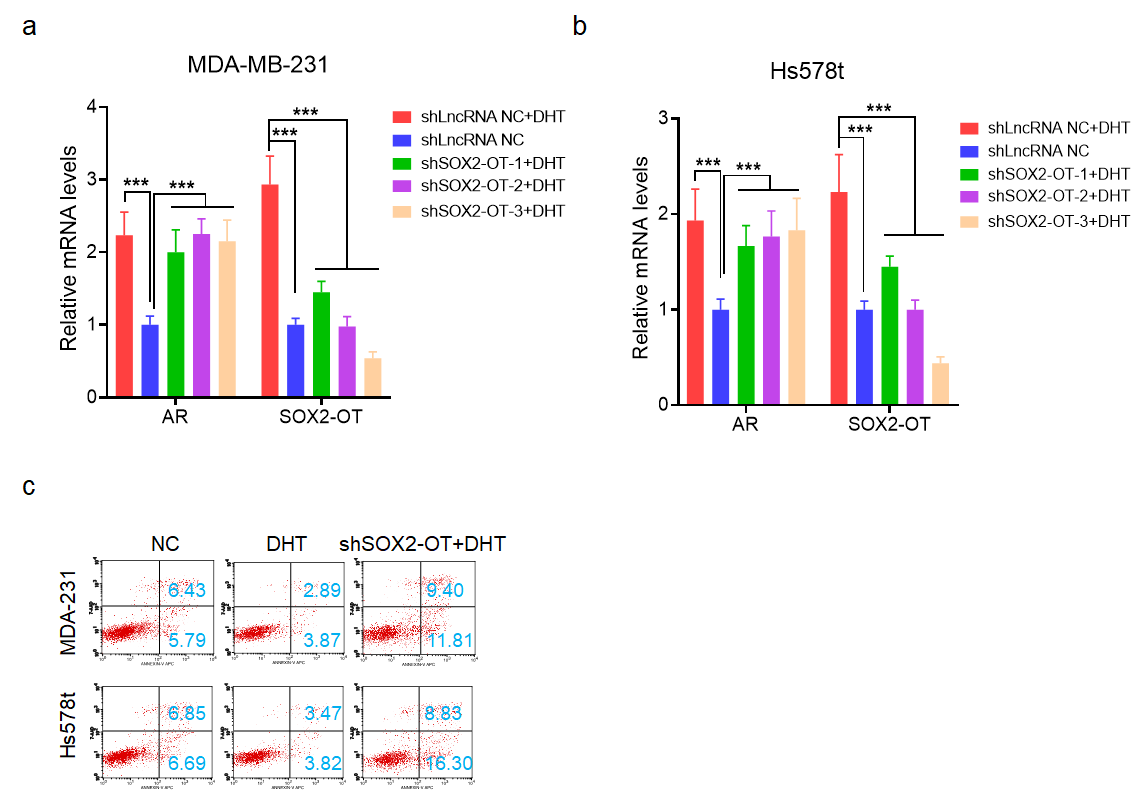
**

**Figure S2, related to Figure 2. AR induces TNBC cells tumorigenesis via SOX2-OT.** a-b) Relative mRNA expression levels of AR and SOX2-OT in the MDA-MB-231 and Hs578t cells treated with three shSOX2-OTs or DHT. ****P* < 0.001 by t test. b) Cell apoptosis assay of MDA-MB-231 and Hs578t cells that were treated with shSOX2-OT or DHT. Results represented the average of three independent experiments, the data represent the mean ± SD.

**Table S1 Different expressed lncRNAs in the** microarray

| **Gene Symbol** | **Fold Change** | **Regulation** | **P-value** | **MDA-231+DHT-1** | **Hs578t+DHT-1** | **MDA-231** | **Hs578t** |
| --- | --- | --- | --- | --- | --- | --- | --- |
| XLOC_007020 | 6.0437905 | up | 0.0494 | 82.97074 | 179.58273 | 31.920668 | 20.536133 |
| FLJ12334 | 3.5471975 | up | 0.0178 | 139.26039 | 137.94289 | 58.14721 | 40.934765 |
| LINC00349 | 2.5911499 | down | 0.0144 | 830.7666 | 856.5731 | 2644.5366 | 2934.502 |
| RP11-379I19.3 | 2.5560762 | up | 0.0288 | 18.6334 | 16.769194 | 8.054389 | 10.55984 |
| SLC7A11-AS1 | 2.4922314 | down | 0.0434 | 4.9999995 | 4.9999995 | 17.759216 | 12.533391 |
| RP11-354M20.3 | 2.4684025 | up | 0.0071 | 19.752249 | 25.586914 | 12.06492 | 11.46343 |
| PTPRVP | 2.4546852 | up | 0.0425 | 5236.85 | 3425.2576 | 2112.6401 | 2237.6897 |
| AC002064.4 | 2.4492751 | up | 0.0072 | 17.165766 | 21.52108 | 10.636119 | 10.01278 |
| RP11-455B3.1 | 2.3954046 | down | 0.0359 | 4.9999995 | 4.9999995 | 16.627052 | 12.368329 |
| RP11-541F9.1 | 2.3138995 | up | 0.0145 | 43.642933 | 49.027508 | 22.481537 | 27.325686 |
| XLOC_000916 | 2.2658915 | up | 0.0084 | 120.542435 | 144.03734 | 77.7388 | 68.27331 |
| CTC-558O2.1 | 2.2628145 | up | 0.0155 | 28.24241 | 28.374542 | 14.588159 | 17.24196 |
| XLOC_005376 | 2.1792603 | up | 0.0154 | 640.58954 | 554.1812 | 376.16916 | 325.53745 |
| SCEL-AS1 | 2.1701306 | up | 0.0127 | 26.090807 | 30.924316 | 17.881958 | 15.41183 |
| AC002127.4 | 2.1478307 | up | 0.0295 | 36.16947 | 43.39351 | 20.190346 | 26.045977 |
| AC007970.1 | 2.1240076 | up | 0.0185 | 43.138878 | 55.33362 | 26.23689 | 30.846794 |
| XLOC_004475 | 2.122785 | up | 0.0449 | 36.481075 | 56.580692 | 25.032814 | 28.06418 |
| SOX2-OT | 2.1117912 | up | 0.0434 | 55.87777 | 94.0361 | 27.943789 | 65.129875 |
| CD99P1 | 2.0269898 | down | 0.0388 | 8.223478 | 11.517648 | 23.510704 | 27.1412 |
| LINC00353 | 2.0106705 | up | 0.0024 | 27.60238 | 32.15819 | 17.917248 | 19.362589 |
| XLOC_003778 | 1.9965663 | down | 0.0107 | 11.22957 | 10.893861 | 27.294725 | 29.2465 |
| RP11-14C22.4 | 1.9755491 | up | 0.0347 | 31.319242 | 29.237974 | 17.717672 | 20.86588 |
| RP11-815J21.4 | 1.9477425 | down | 0.0299 | 12.81457 | 11.106658 | 30.756037 | 28.773546 |
| TET2-AS1 | 1.9216188 | up | 0.0196 | 26.114584 | 30.194214 | 20.07765 | 17.01448 |
| LINC00331 | 1.9182816 | up | 0.0280 | 21.543 | 24.609434 | 13.523078 | 17.14205 |
| RP11-84C10.4 | 1.9147776 | up | 0.0466 | 40.231087 | 38.514893 | 22.69646 | 28.508442 |
| RP11-91H12.3 | 1.9138325 | down | 0.0017 | 10.998501 | 12.826591 | 29.35968 | 28.683218 |
| LOC100507629 | 1.9029518 | up | 0.0377 | 760.9537 | 607.2008 | 478.15002 | 439.68365 |
| RP11-552D8.1 | 1.8948021 | up | 0.0007 | 21.633425 | 24.874247 | 15.626849 | 15.479837 |
| LINC00511 | 1.8726871 | up | 0.0042 | 28.24313 | 33.344704 | 19.677088 | 21.453966 |
| XLOC_010799 | 1.869805 | up | 0.0439 | 31.290894 | 33.455738 | 18.922098 | 24.51467 |
| RP11-265M18.2 | 1.8665552 | up | 0.0434 | 52.08343 | 52.229046 | 39.10361 | 30.428162 |
| RP11-444I9.4 | 1.861547 | down | 0.0141 | 21.42978 | 28.452358 | 56.714207 | 57.52389 |
| RP11-829H16.3 | 1.8585845 | down | 0.0039 | 31.68249 | 39.28175 | 83.47767 | 80.62901 |
| RP11-483I13.4 | 1.8537606 | up | 0.0282 | 20.083961 | 22.212685 | 15.92573 | 13.4192 |
| RP11-536P6.3 | 1.8506334 | up | 0.0201 | 19.209291 | 26.174631 | 15.41614 | 15.339618 |
| LINC00301 | 1.8402769 | up | 0.0046 | 24.576277 | 28.793125 | 18.760578 | 17.67071 |
| RP11-552M11.8 | 1.834752 | down | 0.0093 | 18.22849 | 18.22499 | 43.255154 | 40.96638 |
| RP11-124A7.2 | 1.8307029 | up | 0.0040 | 34.548676 | 39.94258 | 24.301605 | 26.121368 |
| RP11-566K19.3 | 1.8291025 | up | 0.0357 | 29.428465 | 29.646328 | 22.17863 | 18.58972 |
| AC106786.1 | 1.8182415 | up | 0.0232 | 20.659725 | 28.22578 | 16.14179 | 17.392015 |
| CTD-2247C11.3 | 1.8012582 | up | 0.0265 | 39.57248 | 43.50548 | 25.848488 | 31.241806 |
| AC124944.3 | 1.7997304 | down | 0.0499 | 189.27983 | 255.87396 | 565.8978 | 449.6276 |
| ST7OT2 | 1.7806372 | up | 0.0171 | 19.88795 | 23.59682 | 14.25715 | 16.772552 |
| CTA-150C2.13 | 1.7795925 | up | 0.0479 | 29.683949 | 43.23297 | 26.547796 | 23.753824 |
| CTD-2130F23.1 | 1.778202 | up | 0.0465 | 20.283365 | 25.374182 | 14.246449 | 18.283653 |
| XLOC_002792 | 1.7563226 | up | 0.0350 | 20.943209 | 18.828667 | 14.461158 | 14.695412 |
| XLOC_007807 | 1.7560307 | up | 0.0433 | 31.263199 | 32.396637 | 20.313059 | 25.116055 |
| AC016745.3 | 1.7451046 | down | 0.0098 | 34.005215 | 37.799393 | 83.49663 | 72.6977 |
| SEC61A2 | 1.7355085 | down | 0.0258 | 27.982153 | 26.899242 | 59.625248 | 59.233578 |
| FREM2-AS1 | 1.7311989 | up | 0.0429 | 422.0814 | 449.56696 | 376.91364 | 273.81277 |
| RP11-356I2.1 | 1.725892 | up | 0.0079 | 118.2028 | 121.534325 | 84.537964 | 89.11747 |
| XLOC_005832 | 1.7204119 | up | 0.0354 | 48.581623 | 56.292667 | 34.00716 | 41.337166 |
| CTD-2049O4.1 | 1.7125518 | up | 0.0156 | 22.236897 | 26.018435 | 18.70953 | 16.77721 |
| CTBP1-AS1 | 1.7099521 | down | 0.0194 | 25.873713 | 32.56905 | 66.55472 | 57.72259 |
| RP11-540O11.7 | 1.7015855 | down | 0.0335 | 61.796486 | 59.485374 | 123.42544 | 133.35713 |
| ZEB2-AS1 | 1.6969341 | up | 0.0191 | 43.880028 | 50.260025 | 31.906755 | 36.58256 |
| RP11-434I12.3 | 1.6956893 | down | 0.0270 | 15.679799 | 14.695181 | 31.80737 | 33.32117 |
| RP11-231E4.4 | 1.6954895 | down | 0.0443 | 13.287012 | 11.720128 | 27.979712 | 26.023027 |
| RP11-80F22.9 | 1.6949775 | up | 0.0399 | 25.019234 | 30.005856 | 18.190418 | 22.578327 |
| RP11-351A20.1 | 1.6925552 | up | 0.0328 | 20.027378 | 22.823729 | 14.455629 | 17.762838 |
| RP11-753B14.1 | 1.6905774 | up | 0.0204 | 35.86056 | 35.307457 | 25.828367 | 26.317696 |
| RP11-142O6.1 | 1.6762214 | up | 0.0162 | 352.0414 | 327.9678 | 277.1188 | 240.70644 |
| FRMPD3-AS1 | 1.6731069 | up | 0.0450 | 21.972263 | 20.98101 | 17.366518 | 15.395291 |
| RP11-318K15.2 | 1.6721916 | down | 0.0210 | 43.26575 | 42.33825 | 89.07668 | 89.30715 |
| PVT1 | 1.6641827 | down | 0.0114 | 16.03786 | 17.18734 | 36.82667 | 32.945583 |
| XLOC_010348 | 1.6611547 | up | 0.0468 | 185.41739 | 161.96477 | 127.106285 | 135.48961 |
| XLOC_005424 | 1.6603677 | up | 0.0196 | 37.617714 | 48.333405 | 30.808187 | 32.950615 |
| NBPF7 | 1.6595866 | down | 0.0445 | 157.4143 | 197.86383 | 359.5302 | 380.5196 |
| RP4-798C17.6 | 1.6476439 | down | 0.0331 | 12.748809 | 13.20846 | 29.864183 | 25.015066 |
| XLOC_010855 | 1.6312771 | down | 0.0297 | 11.4079895 | 11.587979 | 22.364552 | 25.93908 |
| XLOC_001356 | 1.6279845 | up | 0.0151 | 2500.3333 | 2501.112 | 1980.5481 | 1973.9058 |
| ATXN8OS | 1.6278717 | up | 0.0193 | 21.838617 | 28.409855 | 19.667418 | 18.82313 |
| HMlincRNA858 | 1.6157146 | up | 0.0476 | 22.65754 | 31.94415 | 19.993647 | 21.82309 |
| LOC100190939 | 1.6108823 | up | 0.0033 | 84.67694 | 98.46842 | 72.331696 | 69.17269 |
| AF157115 | 1.6073755 | down | 0.0451 | 629.06146 | 791.13544 | 1475.7534 | 1428.5588 |
| RP11-598C10.2 | 1.6053325 | up | 0.0409 | 52.377613 | 53.10007 | 37.65193 | 43.647434 |
| CTB-131B5.5 | 1.603585 | down | 0.0212 | 24.14829 | 24.16541 | 49.66917 | 47.016747 |
| AC007879.7 | 1.6008911 | down | 0.0332 | 22.602003 | 30.959301 | 52.291954 | 52.857025 |
| LINC00535 | 1.5936322 | up | 0.0329 | 19.608788 | 19.124119 | 16.204412 | 15.02725 |
| XLOC_002197 | 1.5928584 | up | 0.0471 | 151.41591 | 132.94469 | 117.26042 | 106.33027 |
| RP11-501J20.2 | 1.5851746 | up | 0.0474 | 21.05592 | 21.314066 | 18.292418 | 15.748501 |
| XLOC_004677 | 1.5781496 | up | 0.0250 | 5187.3438 | 4586.1743 | 4380.822 | 3375.2073 |
| RP11-588G21.2 | 1.566629 | up | 0.0022 | 18.691202 | 20.251225 | 15.70238 | 15.948121 |
| XLOC_011520 | 1.565729 | up | 0.0429 | 96.74039 | 117.91771 | 79.79283 | 91.40899 |
| XLOC_014066 | 1.5625465 | up | 0.0353 | 1001.92004 | 1157.498 | 862.96234 | 885.5378 |
| XLOC_008372 | 1.5572985 | up | 0.0361 | 15.629912 | 19.785946 | 14.244648 | 15.04888 |
| ARHGEF7-IT1 | 1.5460439 | up | 0.0177 | 20.019958 | 21.2201 | 18.123522 | 15.910198 |
| PLGLA | 1.5455049 | up | 0.0355 | 83.83757 | 104.50378 | 204.43184 | 163.95145 |
| XLOC_002443 | 1.5436003 | up | 0.0364 | 16.963692 | 19.568872 | 13.784518 | 16.581747 |
| CRNDE | 1.5418509 | up | 0.0431 | 511.50137 | 528.1254 | 501.88113 | 377.53854 |
| AC034187.2 | 1.5404 | up | 0.0128 | 17.575413 | 21.995892 | 16.310635 | 16.182383 |
| HMlincRNA814 | 1.5397319 | up | 0.0207 | 43.540478 | 52.39955 | 36.476906 | 40.329075 |
| RP11-148K1.12 | 1.5394946 | up | 0.0322 | 49.19753 | 49.22043 | 42.080868 | 37.244686 |
| RP11-7F17.3 | 1.5374966 | down | 0.0462 | 18.871532 | 25.080591 | 44.514515 | 38.890938 |
| AP001043.1 | 1.536696 | up | 0.0236 | 33.21927 | 41.990086 | 31.701889 | 28.730696 |
| XLOC_003837 | 1.5341732 | up | 0.0033 | 62.55821 | 66.94455 | 53.041237 | 51.644615 |
| XLOC_011938 | 1.5304598 | down | 0.0213 | 30.319809 | 37.144283 | 68.55839 | 60.098873 |
| HERC2P2 | 1.5094871 | down | 0.00960 | 50.765995 | 60.737865 | 109.0659 | 100.284805 |
| RP5-1114G22.2 | 1.5078951 | up | 0.0398 | 25.928253 | 33.27805 | 25.81898 | 22.83784 |
| RP11-325B23.2 | 1.5078119 | down | 0.0427 | 12.07618 | 15.844822 | 27.452631 | 25.435883 |
| XLOC_008599 | 1.507431 | up | 0.0451 | 21.067968 | 20.93204 | 16.451153 | 18.88129 |
| LOC100130000 | 1.5049535 | down | 0.0236 | 26.102701 | 33.94865 | 57.47594 | 54.04037 |
| MTOR-AS1 | 1.5035704 | down | 0.0484 | 21.009857 | 20.479853 | 36.933247 | 40.890503 |
